# EBMT—NIH—CIBMTR Task Force position statement on standardized terminology & guidance for graft-versus-host disease assessment

**DOI:** 10.1038/s41409-018-0204-7

**Published:** 2018-06-05

**Authors:** Helene M. Schoemans, Stephanie J. Lee, James L. Ferrara, Daniel Wolff, John E. Levine, Kirk R. Schultz, Bronwen E. Shaw, Mary E. Flowers, Tapani Ruutu, Hildegard Greinix, Ernst Holler, Grzegorz Basak, Rafael F. Duarte, Steven Z. Pavletic, EBMT (European Society for Blood and Marrow Transplantation) Transplant Complications Working Party and the “EBMT−NIH (National Institutes of Health)−CIBMTR (Center for International Blood and Marrow Transplant Research) GvHD Task Force”

**Affiliations:** 1 Department of Hematology, University Hospital Leuven and KU Leuven, Leuven, Belgium; 2 Clinical Research Division, Fred Hutchinson Cancer Research Center, Seattle, WA, USA; 3 Division of Hematology and Medical Oncology, Tisch Cancer Institute, Icahn School of Medicine at Mount Sinai, New York, NY, USA; 4 Department of Internal Medicine III, University Hospital Regensburg, Regensburg, Germany; 5 Michael Cuccione Childhood Cancer Research Program, BC Children’s Hospital, UBC, Vancouver, Canada; 6 Center for International Blood and Bone Marrow Transplant Research (CIBMTR), Medical College of Wisconsin, Milwaukee, WI, USA; 7 Clinical Research Institute, Helsinki University Hospital, Helsinki, Finland; 8 Division of Hematology, Medical University of Graz, Graz, Austria; 9 Department of Hematology, Oncology and Internal Medicine, Medical University of Warsaw, Warszawa, Poland; 10 Hospital Universitario Puerta de Hierro Majadahonda, Madrid, Spain; 11 Center for Cancer Research National Cancer Institute, National Institutes of Health, Bethesda, MD, USA

## Abstract

Several international recommendations address the assessment of graft-versus-host disease (GvHD) after hematopoietic cell transplantation (HCT). This position statement by GvHD experts from the European Society for Blood and Marrow Transplantation (EBMT), the National Institutes of Health (NIH) and the Center for International Blood and Marrow Transplant Research (CIBMTR) reviews the existing guidelines for both acute and chronic GvHD, addresses potential confusions that arise in daily practice and proposes consensus definitions for many key terms. We provide a historical perspective on the currently available guidelines and recommend the Mount Sinai Acute GvHD International Consortium (MAGIC) criteria for acute GvHD and the NIH 2014 criteria for chronic GvHD as the most comprehensive and detailed criteria available. This statement also offers practical guidance for the implementation of these recommendations and a set of consensus definitions for commonly used GvHD terms in order to facilitate future clinical and translational research. To assist the dissemination of these recommendations, a web-application based on this position statement is available (https://www.uzleuven.be/egvhd). We believe that adherence to a common set of GvHD assessment criteria is vitally important to improve the quality of data, compare results of retrospective studies and prospective clinical trials, and make therapeutic recommendations based on quality evidence.

## Introduction

Graft-versus-host disease (GvHD) refers to a clinical syndrome caused by the response of transplanted donor allogeneic cells to histocompatibility antigens expressed on tissues of the transplantation recipient. It is the most serious complication of allogeneic hematopoietic cell transplantation (HCT). Its recognition and control are key elements of a successful outcome. In fact, the World Health Organization stipulates that data collection and data analysis are integral parts of therapy [1].

In practice, however, the application of basic concepts pertaining to the diagnosis and staging of this condition differs widely among HCT clinicians. The use of the templated data collection forms (such as those used by the Center for International Blood and Marrow Transplant Research (CIBMTR), the European Society for Blood and Marrow Transplantation (EBMT), and the National Institutes of Health/National Cancer Institute (NIH/NCI)) improves standardization by collecting data elements as proposed by published consensus documents but demands significant time from healthcare professionals and researchers.

Several studies have shown a lack of adherence to recommendations and inconsistencies in GvHD evaluation [2–9]. Weisdorf et al. showed in one multi-center study that acute GvHD (aGvHD) grading at HCT centers significantly underestimated disease severity compared to a central, expert review board, with inaccurate evaluation of grade III GvHD in 18% of cases [7]. In a recent chronic GvHD (cGvHD) intervention trial, up to 10% of patients entered by GvHD Consortium centers were excluded from study analysis post hoc due to failure to meet diagnostic criteria at the time of inclusion [8].

Such discrepancies are concerning because they can significantly affect the interpretation of GvHD data in clinical trials. Misclassifications have been observed even among experienced HCT and GvHD professionals, and inaccuracies are therefore likely to be even more prominent among less experienced centers. In fact, a recent survey of practice patterns completed by transplant professionals during the annual 2017 EBMT conference showed wide variations in the types of reference guidelines used for GvHD assessments, and up to one third of the survey participants reported a lack of confidence in their ability to apply these guidelines [9]. Of interest, the GvHD assessments of two clinical vignettes became much more consistent and compliant with recent international guidelines when the same cases were evaluated using an electronic tool, the eGvHD App [9] (available at https://www.uzleuven.be/egvhd).

The use of electronic tools to streamline and increase the reliability of the GvHD evaluation process has been advocated by several groups [4, 9–12], but such tools require a clear and broad consensus regarding reference guidelines to guarantee their internal validity. GvHD experts from the EBMT, NIH, and CIBMTR have therefore joined forces to: (1) review the existing guidelines for both acute and chronic GvHD and recommend those best supported by clinical evidence; (2) address confusions that arise in real-life scenarios encountered in clinical practice; and (3) develop consensus definitions for key terms frequently used in the evaluation and monitoring of GvHD. All three issues were addressed during a series of conference calls and manuscript draft reviews between May and October 2017. The mission of this effort is to advance GvHD research through a transparent and unbiased standardization of common elements in GvHD terminology, thereby increasing the quality and precision of the data collected in HCT clinical research and practice.

## Issue 1: Standardized assessments of GvHD: a historical perspective

### Acute GvHD definition

**Fig. 1:**
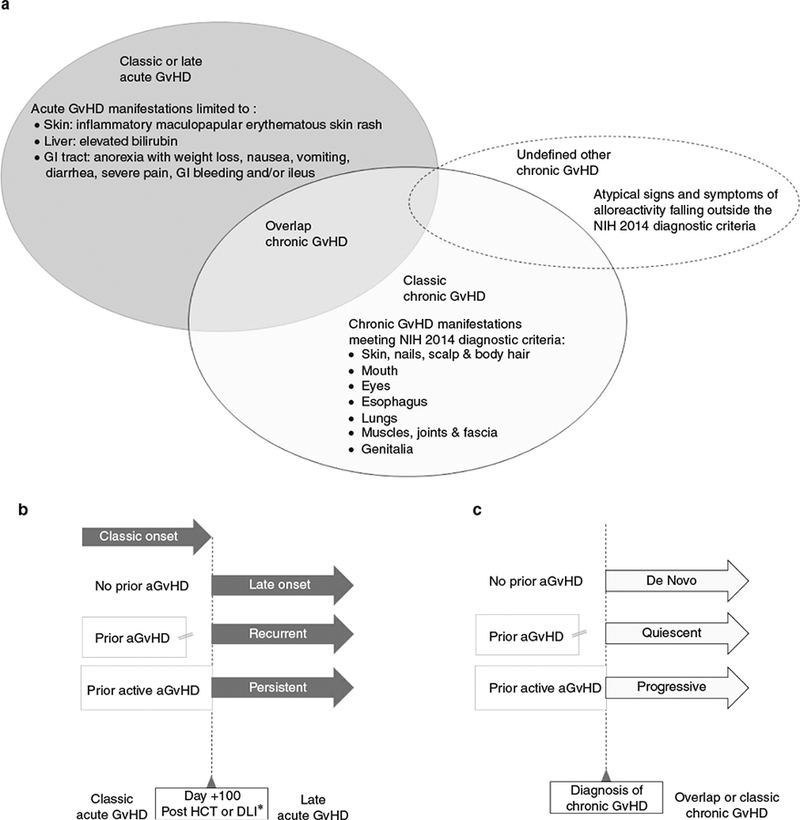
Schematic representation of the types of GvHD and their onset: a Types of GvHD; b Types of acute GvHD onset and c Types of chronic GvHD onset. DLI donor lymphocyte infusion, GvHD graft versus host disease, GI gastro-intestinal tract, HCT hematopoietic cell transplantation, ≠ Controlled, inactive or resolved, * whichever happened last, Δ GvHD onset.

Acute GvHD refers to the appearance of an allogeneic inflammatory response in exclusively three organs: the skin (inflammatory maculopapular erythematous skin rash), the liver (hyperbilirubinemia due to cholestatic jaundice), and the gastro-intestinal (GI) tract (upper and/or lower GI tract manifestations: anorexia with weight loss, nausea, vomiting, diarrhea, severe pain, GI bleeding and/or ileus) [13–16]. The diagnosis must occur in the absence of manifestations of cGvHD [17, 18] (Fig. 1a) and should ideally be supported by positive histological findings, but this is not strictly necessary if no alternative etiology is present.

The Glucksberg aGvHD classification was first proposed in the 1970s based on a cohort of 60 patients evaluated for aGvHD after myeloablative conditioning. This classification staged skin, lower gastrointestinal tract and liver, each on a scale of 0 (absent) to 4 (severe) points (Table 1), to create a final overall grade of I (mild) to IV (life-threatening) [13]. The overall aGvHD grade typically corresponds to the highest grade conferred by the individual staging of each organ, as described in Table 2. Approximately 20 years later, the Keystone aGvHD consensus panel reviewed the outcome of the Glucksberg classification in almost 6000 patients and confirmed the predictive value of maximum aGvHD grade for day 100 mortality [14]. Three major recommendations that resulted from that review were: (1) upper GI tract manifestations, in the presence of a positive biopsy, should be classified as overall grade II aGvHD; (2) GI stage 4 should be based on severe symptoms such as severe pain, bleeding and/or ileus and not diarrhea volume; and (3) functional status should be eliminated as an element of overall grade because of its non-specific and multi-factorial etiology. In parallel, the CIBMTR proposed the IBMTR aGvHD classification: this alternative algorithm was based on similar raw organ staging (Table 1) and resulted in a final grade of A–D (Table 2), which provided a slightly more accurate prediction of mortality [15]. Recently, MacMillan and colleagues published a further adaptation of the Keystone consensus criteria: the Minnesota aGvHD grading, which limited overall grade IV aGvHD to skin and gut stage four, instead of skin and liver stage four as described in the Keystone criteria [19] (Table 2). In this study, no particular grading system was superior in predicting survival. The availability of these different options to assess aGvHD can give rise to controversy when healthcare professionals do not clearly define which grading system is used.

Most recently, the Mount Sinai Acute GvHD International Consortium (MAGIC) has revisited these criteria based on a review of their extensive database containing detailed clinical information on aGvHD, and recommended more precise definitions for grade IV aGvHD [16]. Specifically, stage 4 cutaneous involvement requires the presence of ulcerations or bullous formations on a minimum of 5% of the body surface area. Stage 4 lower GI aGvHD is also considered an overall grade of IV, better reflecting its dismal prognosis [20]. Guidance for the classification of GI involvement is given with thresholds for both upper GI (based on a minimum number of precisely defined symptoms, with or without a positive biopsy) and lower GI tract (based on the number of liquid stool episodes and/or average volume per episode) (Table 1). The MAGIC criteria are actively used by several international consortia (the BMT Clinical Trials Network and the Children’s Oncology Group) and in biomarker development research. In the opinion of this panel, the MAGIC criteria are considered the most current and detailed criteria to diagnose and score the severity of aGvHD, especially for the clarity of what constitutes clinically significant upper GI symptoms and stage 4 skin and GI involvement. It should be noted that there is little difference anticipated between the MAGIC and modified Glucksberg criteria when grades III and IV are combined for analysis. The changes in the definition of upper GI GVHD could affect assignment to overall grades I or II.

Of note, the MAGIC group also introduced the concept of diagnostic confidence levels for acute GvHD: “confirmed”, “probable”, “possible” and “negative” correlating with histological confirmation, initiation of treatment, resolution without therapeutic intervention, and definitive alternative histologic diagnosis, respectively. Further prospective validation of the confidence categories is underway to formally assess their predictive value and reliability.

### Chronic GvHD definition

Chronic GvHD was originally defined in the early 1980s in a cohort of 20 Seattle patients, as any GvHD present beyond day 100. cGvHD severity was categorized as “limited” (localized skin lesions with or without limited hepatic involvement) or “extensive” (generalized skin involvement, major hepatic complications, or involvement of any other organ) [21]. 20 years later, a survey of transplant professionals’ responses to clinical cGvHD vignettes demonstrated wide variations in scoring practices [3] and led to a refinement of the original Seattle criteria (Table 3) [22].

In 2005, the first NIH “expert-opinion” consensus conference for cGvHD defined precise criteria for the diagnosis and staging of individual organ severity, based on functional disability, and eliminated the requirement that all GvHD occurring after day 100 be considered cGvHD [17]. The conference proposed that the diagnosis of cGvHD rely on either specific diagnostic signs or other distinctive signs accompanied by additional *confirmation* (e.g. biopsy or other objective diagnostic test) in at least one target organ (skin and appendages, mouth, eyes, genitalia, esophagus, lungs and muscles and fascia). The “overlap cGvHD sub-type” was defined by the diagnosis of cGvHD together with acute GvHD manifestations of the skin, liver or gut (Fig. 1a). The severity of cGvHD (either classic or overlap) was scored by patient symptoms as well as functional organ impairment, ranging from 0 (absent) to 3 (severe) for each involved organ. A final global severity score for cGvHD is “mild” when a maximum of two organs are scored 1, “severe” if any organ is scored 3, and “moderate” for all other combinations. Lungs provide the single exception to this rule, where a lung score of 1 results in a global score of “moderate”, and a lung score of 2 results in an overall “severe” score because of the potential irreversibility of pulmonary lesions and the poor prognosis for patients so affected [23, 24].

In 2014, a second NIH consensus conference revisited and updated these criteria based on the evidence generated during the intervening decade [18]. One major recommendation was to eliminate from the severity score any dysfunction unequivocally caused by an alternative etiology. Several further refinements to single organ staging were also recommended. In the opinion of this task force, the NIH 2014 criteria are the most accurate and widely accepted standard for the diagnosis and scoring of cGvHD.

## Issue 2: Application to clinical practice

Because the above-mentioned guidelines were developed for research purposes, their application to “real-life” scenarios can be quite challenging for healthcare professionals. This section offers guidance for the application of these international standards in clinical practice.

### Assessment of the global severity of GvHD

The patient’s global severity assessment (overall grade) evaluates exclusively three organs for aGvHD (skin, liver, and GI tract) and eight organs for cGvHD (skin, mouth, eyes, GI tract, liver, lungs, muscles/joints/fascia and genitals), based on the highest score of organ involvement as described above (Tables 2 and 3). No other abnormalities have an impact on the global severity scoring. The patient’s functional status is documented by Karnofsky−Lansky scores, but it does not contribute to the overall score of either acute [14–16, 19] or chronic GvHD [17, 18]. Similarly, “undefined other” cGvHD manifestations or the “opinion of the evaluator” should be recorded but should not have an impact on the final global score [18].

### Multiple causes of organ impairment

For both acute and chronic GvHD, a given organ is not considered in the overall GvHD grade if the manifestation is solely due to a non-GvHD cause (e.g. zoster skin infection, chronic obstructive pulmonary disease, steroid myopathy, etc…). In the case of both GvHD and concomitant non-GvHD etiologies, it is useful to document the non-GvHD causes but there is currently no justification to downgrade an organ score due to concurrent additional causes (e.g. simultaneous liver GvHD and veno-occlusive disease) [18, 25].

### Organ-specific issues

Acute GvHD typically only involves three organs: the skin, the liver, and the GI tract [16]. Alloimmune manifestations in other organs are to be linked to chronic GvHD (Fig. 1a) [18]. For instance, oral GvHD with lichen planus-like changes is always considered to be a chronic manifestation even if it appears in the early post-transplantation phase (where it needs to be differentiated from alternative etiologies). Obstructive lung manifestations are also always considered to be chronic features, provided they are either confirmed by biopsy or meet strict diagnostic criteria and are accompanied by at least one diagnostic or distinctive manifestation of cGvHD elsewhere [18].

Some patients have atypical signs and symptoms that might be considered cGvHD but fall outside of the current diagnostic, staging and response criteria [18, 27]. Such manifestations of potential alloreactivity (e.g. ascites, serositis, nephrotic syndrome, membranous glomerulopathy, myasthenia gravis, peripheral neuropathy, polymyositis, weight loss in the absence of GI symptoms, Raynaud’s phenomenon, cardiac involvement, eosinophilia, decreased platelet counts, thyroid disorders, etc…) [18] can occur at any time after transplantation. If attributed by the treating physician to cGvHD, they should be categorized as “undefined other cGvHD” (Fig. 1a). This category may represent 10–15% of patients (Kirk Shultz, personal communication). Capturing these data in prospective cohorts is recommended to understand the full spectrum and true incidence of immunological complications after HCT, especially when such manifestations drive management decisions (e.g. the treating physician alters immunosuppression suspecting a link with cGvHD). All manifestations treated as cGvHD should thus be documented, irrespective of whether they meet NIH diagnostic criteria, provided that their “undefined other” nature is clearly noted.

Similarly, isolated increase of transaminases is relatively common during the taper of immunosuppression or after donor lymphocyte infusions. This increase should also be assigned to the “undefined other cGvHD” group, provided it is treated as GvHD in the absence of meeting NIH diagnostic criteria and no histopathological confirmation of liver GvHD has been obtained. Because of their invasive character, liver biopsies are rarely performed and the nature of hepatic enzyme disturbances remains therefore uncertain. This further emphasizes the need for prospective recording of such abnormalities [28].

### Overlap chronic GvHD

Overlap cGvHD is a subtype of cGvHD which has been associated with a poor prognosis [29, 30]. It is characterized by the simultaneous presence of acute and chronic GvHD features (Fig. 1a). Chronic GvHD that is accompanied by acute GI manifestations (anorexia, nausea, vomiting, diarrhea, severe abdominal pain, GI bleeding, and/or ileus) is categorized as overlap cGvHD [17, 18]. However, skin manifestations of aGvHD (maculopapular erythematous rash) can be difficult to differentiate from those of cGvHD. Similarly, the elevation of bilirubin (often accompanied by elevated hepatic enzymes) suggests involvement of the liver, but cannot be unequivocally attributable to either an acute or a chronic process. Given these uncertainties, we currently recommend systematic documentation of aGvHD manifestations (in any organ) and subclassification of such cases as overlap cGvHD, while awaiting future “biology-based” classifications.

### Specific guidance for the assessment of chronic GvHD

#### Skin, muscle, and fascia involvement

In cGvHD, MRI can sometimes be a useful tool to detect fascia involvement [31], yet distinguishing between skin and muscle/fascia fibrosis as the cause of functional impairment is frequently challenging. Once movement is impaired, muscles and fascia are generally involved and are almost always associated with sclerotic skin GvHD [32]. Therefore, skin and fascia involvement should then be documented, even if skin involvement is the primary manifestation. Furthermore, although photographic-range of motion (P-ROM) ratings have been recognized as a sensitive way to capture fascia involvement and response to treatment [33], they cannot be directly translated into severity scores of joints-fascia involvement [18]. Finally, muscle cramps are frequently reported by GvHD patients but are not specific and are not included in the severity score.

#### Scheduling pulmonary function tests and genital exams

Clinical practice rarely allows time and resources for an exhaustive patient evaluation of cGvHD at every visit. For example, pulmonary function tests (PFTs) and genital examinations typically require third-party input, which can be challenging to obtain on the same day.

Although both the dyspnea and lung function scores should ideally be recorded, PFTs are the best way to describe lung involvement and should be obtained at diagnosis of GvHD and then minimally every 3–6 months thereafter in patients on systemic therapy for active cGvHD [34, 35]. However, if recent (maximum 3–6 months old) PFTs are missing, we recommend that symptomatic dyspnea score be used for scoring [18] until updated PFTs are available. Documentation should ideally allow tracing of which source of information (symptoms or PFTs) was used, to allow for meaningful comparisons over time.

A formal genital exam or inspection should ideally be performed at diagnosis and at every GvHD evaluation thereafter in patients with active cGvHD. In clinical practice, this is not always feasible; therefore, we recommend this exam be performed within 3 months of cGvHD diagnosis followed by a regular follow-up every 9−12 months [35, 36]. At other time points, a genital exam is recommended when a patient reports specific discomfort or new lesions in the genital area.

Of note, both pulmonary and urogenital complications can go undetected if not specifically queried, with potentially dramatic clinical consequences [23, 24, 37–39]. Patients should be asked about symptoms and functional impairments at every visit, since early recognition of these complications can often be addressed with relatively simple therapeutic measures, including local or limited systemic immunosuppressive treatment [36, 40, 41].

#### Controversies in chronic GvHD

In spite of the extensive harmonization effort of the cGvHD NIH consortium, some criteria would benefit from further clarification. For instance, weight loss is categorized based on the percentage decrease of bodyweight occurring over a 3-month period [18]. It is unclear how to classify patients who lose a significant amount of weight initially but have stabilized by the time of evaluation. For now, we recommend to limit the impact of weight loss on severity scoring to the last 3 months preceding the GvHD assessment time point. Another controversial issue is the use of therapeutic measures to define severity (e.g. the placement of punctal plugs for severely dry eyes [18, 27], the use of specific eye ware to relieve pain [18, 27] or the dilatation of esophageal stenosis [18]). Given the lack of empirical data, clarification of these issues will require consensus and validation efforts in the future. In the meantime, we recommend to track therapeutic interventions and specify in clinical protocols and/or standard operating procedures whether the severity score considers treatments/procedures ever received or within a specific timeframe.

### Pediatric considerations

Three primary areas differ in the pediatric population with regards to GvHD assessment: (1) some criteria used in adults are difficult to apply in young children (e.g. PFTs and Schirmer’s test for children under the age of 6 [18]); (2) the incidence of cGvHD appears lower in children [42, 43]; and (3) approximately 50% of pediatric transplants are performed for nonmalignant disorders, where tissue repair defects that may impact development of GvHD are more common (e.g. increase of aGvHD in Fanconi Anemia patients [44]).

Currently, the only organs with specific pediatric modifications recommendations for GvHD assessment are: (1) adapted body surface area maps for skin involvement; (2) appropriate reference values for lung function; and (3) weight-adapted measures for diarrhea [16, 18]. Moreover, as PFTs are unreliable for children under the age of 6 years, diagnosis and scoring of lung GvHD relies instead on clinical evaluation, imaging, and lung biopsy [18]. The high frequency of usually transient viral erythema, which can be mistaken for manifestations of aGvHD, is another issue in children. There is thus clearly an unmet need for developing pediatric population-adapted GvHD symptom scales and assessments [45].

## Issue 3: A standardized GvHD terminology

In clinical practice, GvHD presentations can range from a rapidly progressive extensive inflammatory syndrome requiring immediate and aggressive systemic immune suppression, to purely fibrotic, cicatricial manifestations with fixed deficits that are unlikely to respond quickly or completely resolve with therapy [26]. Between these extremes, the large spectrum of presentations, occurring in the context of a wide variety in GvHD prevention and treatment regimens, is more challenging to describe. Many of the terms frequently used to communicate with patients and colleagues lack clear, broadly accepted definitions. We propose here several definitions for a standardized GvHD terminology in order to facilitate future research and allow more accurate comparisons among studies (Table 4).

### GvHD activity

In the setting of clinical trials, response to treatment compares disease burden at specific points in time, usually with regards to a particular treatment. It is based on a number of clinical findings, sometimes including fixed deficits. Classical categories of response are complete response (CR), partial response (PR), and lack of response (which includes no change, mixed response and progression), as established by the NIH consortium for chronic GvHD [27]. For acute GvHD, similar criteria have been described by the MAGIC consortium [46].

However, GvHD activity may be distinct from response if the disease burden includes fixed deficits that are no longer responsive to treatment. Identification of such deficits can be difficult but is essential to the accurate description of complex clinical phenotypes, particularly in cGvHD. Determination of GvHD activity is often the principal driver in therapeutic decisions (e.g. intensification, reduction (taper) or discontinuation of immunosuppression) and is likely to be critical for biomarker validation. We therefore propose a classification of GvHD activity that incorporates both the presence of disease manifestations and the use of immunosuppression, consistent with the NIH Consensus task force model of GvHD physio-pathology [26].

GvHD is considered “clinically active” if the patient has inflammatory or worsening manifestations (either acute or chronic) regardless of the use of immunosuppressive therapy. After the inflammation resolves, GvHD manifestations can either disappear without residua or fixed deficits may remain. Such fixed or irreversible deficits represent scars in the affected organ due to either permanent damage or aberrant tissue repair (e.g. skin color change, stable fibrotic features, sicca syndrome) that persist regardless of immunosuppressive treatment [26].

Once all signs of clinical activity have disappeared, GvHD activity can be described in three different ways. If immunosuppression is still ongoing or has been discontinued for less than 12 weeks [26] or 24 weeks [47] for acute and chronic GvHD respectively, GvHD activity can be considered “controlled” regardless of the presence of fixed sequelae. If immunosuppression has been discontinued for more than the above mentionned periods of time without recurrence of inflammatory signs, GvHD is termed “resolved” if there are no fixed deficits and “inactive” if such fixed deficits persist.

### GvHD onset

GvHD onset refers to the presentation of the first episode of clinically evident alloreactivity of the donor against the recipient host (Fig. 1b, c).

“Classic acute GvHD” refers to the initial diagnosis of acute GvHD within the first 100 days following transplantation or DLI infusion (whichever happened last) [17]. “Late acute GvHD” occurs beyond day 100 and can be: “late onset” (new onset of aGvHD with no prior history of classic aGvHD), “recurrent onset” (recurrence of aGvHD in a patient with prior history of classic aGvHD whose symptoms became controlled, inactive or resolved); or “persistent” if active aGvHD signs persist beyond day 100 in the absence of cGvHD manifestations [17].

Chronic GvHD is referred to as having “de novo onset” if cGvHD is diagnosed [18] for the first time in a patient who did not previously experience acute GvHD [17, 18]. “Quiescent onset” is defined as cGvHD that appears for the first time after all acute GvHD manifestations have become controlled, inactive or resolved [17, 18]. “Progressive onset” refers exclusively to the initial presentation of cGvHD manifestations while acute GvHD symptoms are still active [17, 18]. It is therefore always a form of overlap cGvHD (Fig. 1a), although not all overlap cGvHD syndromes present with a progressive onset. “Progressive onset” is also distinct from “progression”, which is a response criterion that refers to an increase in severity of acute or chronic GvHD symptoms over time [27, 46]. “Progressive onset” cGvHD has been associated for over 30 years with inferior prognosis and poor response to treatment [48–60]. Yet, it should be noted that because these studies used a variety of definitions, some patients, who did not present with new cGvHD manifestations, would now be reclassified as “persistent late acute GvHD”. Interestingly, Stewart et al. showed that after the dose of prednisone was taken into account, “progressive onset” no longer predicted long-term survival [60], suggesting that the level of chronic immunosuppression at diagnosis influences the prognosis for cGvHD with this type of onset.

There is currently no formal nomenclature to refer to the pattern of GvHD recurrence after an initial diagnosis. The term “flare” is sometimes used to define the reappearance or worsening of any signs of GvHD. Although this might reflect the natural course of the disease, this term currently lacks a validated definition. For written scientific communications, we recommend instead the precise terminology that refers to disease onset [17] or the classical clinical trial response criteria [27], as appropriate.

### Response to steroids

Acute GvHD steroid refractoriness or resistance is most often referred to as either (1) progression in any organ within 3 [61–71], 4 [72–76], or 5 [77–79] days of therapy onset with ≥2 mg/kg/day [61–63, 69–71, 73, 74, 76–78, 80–84] of prednisone equivalent, (2) failure to improve within 5 [67] to 7 [61, 62, 64–66, 68, 69, 72, 74–76, 78, 80, 81, 83] days of treatment initiation [71, 79, 85] or (3) incomplete response after more than 28 days of immunosuppressive treatment including steroids [46]. For the determination of eligibility in prospective clinical trials, alternative definitions for aGvHD steroid refractoriness may include other aspects such as: incomplete response after 14 days of therapy [64–66, 75, 78, 79, 86] or use of an additional immunosuppressive agent [86]. Chronic GvHD steroid refractoriness or resistance is typically referred to as either: (1) progression of GvHD while on prednisone at ≥1 mg/kg/day for 1 [87] to 2 [88] weeks; or (2) stable GvHD on ≥0.5 mg/kg/day (or 1 mg/kg every other day) of prednisone for 1 [87, 89] to 2 months [65, 88].

Steroid dependence has been defined for aGvHD as the inability to taper prednisone under 2 mg/kg/day after an initially successful treatment of at least 7 days [74, 80, 81] or as the recurrence of aGvHD activity during steroid taper [68, 79]. The relevance of this term was shown by Martin and colleagues who demonstrated that the highest CR rates with secondary therapy were seen when aGvHD recurred during the taper phase of the primary glucocorticoid treatment, thereby distinguishing it from steroid refractory aGvHD [90]. In cGvHD, steroid dependence refers to the inability to control GvHD symptoms while tapering prednisone below 0.25 mg/kg/day (or 0.5 mg/kg every other day) in at least two individual attempts, separated by at least 8 weeks [87].

Finally, the term “steroid intolerance” has not been formally validated but refers to the emergence of unacceptable toxicity (e.g. uncontrolled infections, avascular necrosis, arterial hypertension, diabetes mellitus, myopathy, osteoporosis, etc.) attributed to corticosteroids, as evaluated by a healthcare professional [91, 92].

## Conclusions

This report stresses the critical importance of a common, international approach to describe the variety of GvHD clinical manifestations observed after HCT. In the era of electronic patient records and e-health applications, it is possible to apply complex algorithms at the bedside and follow internationally vetted guidelines in daily clinical practice. Several efforts in this direction [4, 9–12], such as the eGVHD app (available at https://www.uzleuven.be/egvhd), are already developing more standardized and accurate methods to capture “real-world” GvHD data. This progress underlines the responsibility of transplantation societies to help clarify definitions, to facilitate comparisons of clinical research results and to set standards for clinical practice.

This task force panel advocates the use of the MAGIC criteria for aGvHD and the NIH 2014 criteria for cGvHD as the most comprehensive and detailed criteria currently available. In addition, this statement provides consensus definitions for a lexicon of commonly used GvHD terms and concepts in order to facilitate GvHD clinical research.

The standardization of GvHD assessments should be a dynamic process that can incorporate progress in new diagnostic and therapeutic approaches. Even as refined classifications improve communication among clinicians, they should also be prospectively evaluated for their predictive potential. Furthermore, in the absence of any pathognomonic signs or test for GvHD, subjective elements remain an integral part of the final clinical assessment. As prospective biomarkers that detect underlying GvHD pathophysiology are validated, they may assist clinicians by offering objective laboratory metrics in addition to clinical GvHD manifestations. But the formal validation of these markers requires accurate and reliable clinical assessment of GvHD severity in all organs.

We hope that this position statement will serve as the cornerstone of a larger scale consensus project. Consistent adherence to common sets of criteria, such as those endorsed here, will help the transplantation community to improve the quality of data capture across all types of GvHD manifestations and therapeutic strategies. Harmonization of standards for the accurate assessment of GvHD is an essential prerequisite for the formulation of recommendations [85, 93] regarding GvHD prophylaxis and treatment that are based on quality evidence.

## Figures and Tables

**Table 1:** Comparison of the different guidelines available for acute GvHD assessment: individual organ severity staging

Organ Severity Stage	Original Glucksberg criteria [13]	“Modified Glucksberg” or “Keystone” criteria [14] and IBMTR criteria [15]	MAGIC criteria [16]
Skin			
0	No rash	No rash	No rash
1	Rash <25% of BSA	Rash <25% of BSA	Rash < 25% of BSA
2	Rash 25% to 50% of BSA	Rash 25% to 50% of BSA	Rash 25% to 50% of BSA
3	Rash > 50% of BSA	Rash > 50% of BSA	Rash > 50% of BSA
4	Generalized erythroderma with bullous formation	Generalized erythroderma with bullous formation	Generalized erythroderma (> 50% BSA) plus bullous formation and desquamation >**5% of BSA**
Liver			
0	Total serum bilirubin <34 μmol/L (<2 mg/dL) or AST/SGOT 150–750 IU	Total serum bilirubin <34 μmol/L (< 2 mg/dL)	Total serum bilirubin < 34 μmol/L (< 2 mg/dL)
1	Total serum bilirubin 34–50 μmol/L (2 to 3 mg/dL)	Total serum bilirubin 34–50 μmol/L (2 to 3 mg/dL)	Total serum bilirubin 34–50 μmol/L (2 to 3 mg/dL)
2	Total serum bilirubin 51–102μmol/L (3.1 to 6mg/dL)	Total serum bilirubin 51–102μmol/L (3.1 to 6 mg/dL)	Total serum bilirubin 51–102μmol/L (3.1 to 6 mg/dL)
3	Total serum bilirubin 103–255 μmol/L (6.1 to 15 mg/dL)	Total serum bilirubin 103–255 μmol/L (6.1 to 15 mg/dL)	Total serum bilirubin 103–255 μmol/L (6.1 to 15 mg/dL)
4	Total serum bilirubin > 255 μmol/L (>15 mg/dL)	Total serum bilirubin > 255 μmol/L (> 15 mg/dL)	Total serum bilirubin >255 μmol/L (> 15 mg/dL)
Upper GI			
0	NA	No persistent nausea and no histologic evidence of GvHD in the stomach or duodenum	**No or intermittent**^a^ anorexia or nausea or vomiting
1	NA	Persistent nausea **with histologic evidence of GvHD** in the stomach or duodenum	**Persistent**^a^ anorexia or nausea or vomiting
Lower GI			
0	Diarrhea <500 mL/day	Diarrhea < 500 mL/day	Diarrhea < 500 mL/day or<3 episodes/day for adults^b,c^
1	Diarrhea >500 mL/day	Diarrhea > 500 mL/day	Diarrhea 500–999 mL/day or 3–4 episodes/day for adults^b,d^
2	Diarrhea > 1000 mL/day	Diarrhea > 1000 mL/day	Diarrhea 1000–1500mL/day or 5–7 episodes/day for adults^b,e^
3	Diarrhea > 1500 mL/day	Diarrhea > 1500 mL/day	Diarrhea >1500 mL/day or >7 episodes/day for adults^b,f^
4	Diarrhea >2000 mL/day	Severe abdominal pain with or without ileus	Severe abdominal pain **with or without ileus or grossly bloody stools (regardless of stool volume)**
Karnofsky index			
	>30%	NA	NA
	<30%	NA	NA

*AST* (Aspartate transaminase); *BSA* (Body surface area); *GI* (Gastro-intestinal tract); *GvHD* (Graft versus Host Disease); *IBMTR* (International Bone Marrow Transplantation Registry); *IU* (International units); *MAGIC* (Mount Sinai Acute GvHD International Consortium); *NA* (Not applicable); *SGOT* (Serum glutamic oxaloacetic acid transaminase)

a **To be suggestive for GvHD:** anorexia should be accompanied by weight loss, nausea should last at least 3 days, or be accompanied by at least 2 vomiting episodes per day for at least 2 days [16]

b One episode of diarrhea is considered to be about 200 ml for an adult and 3 ml/kg for a child (< 50 kg) [16]

c Diarrhea <10 mL/kg/day or <4 episodes/day for children

d Diarrhea 10–19.9 mL/kg/day or 4–6 episodes/day for children

e Diarrhea 20–30 mL/kg/day or 7–10 episodes/day for children

f Diarrhea > 30 mL/kg/day or >10 episodes/day for children

**Table 2:** Comparison of the different guidelines available for acute GvHD assessment: overall severity grading

Overall Glucksberg/MAGIC grade	Original Glucksberg criteria [13]	“Modified Glucksberg” or “Keystone” criteria [14]		MAGIC criteria[16]	IBMTR criteria [15]	Overall IBMTR grade
0	no organ involvement (skin=0; and liver=0; and GI=0) corresponds to the absence of aGvHD					0
I	skin=1 or 2, without liver/GI involvement or decrease in performance status/fever		skin = 1 or 2, without liver/GI involvement		skin=1, without liver/GI involvement	A
II	skin=1 or 2 and (liver and/or GI involvement=1 or 2) with mild decrease in performance status		skin=3; and/or liver=1; and/or GI=1		skin=2; and/or liver =1 or 2; and/or GI=1 or 2	B
III^a^	(skin and/or liver and/or GI=2, 3 or 4) with marked decrease in performance status	liver=2 or 3; and/or GI=2, 3 or 4^a^		liver=2 or 3; and/or GI=2 or 3	skin=3; and/or liver=3; and/or GI=3	C
IV^b^	(skin and/or liver and/or GI=2, 3 or 4) with Karnofsky <30%	skin=4; and/or liver=4^b^		skin=4; and/or liver=4; and/or GI=4		D

The overall aGvHD grade typically corresponds to the highest grade conferred by the individual staging of each organ. *GI* (Gastro-intestinal tract); *GvHD* (Graft versus Host Disease); *IBMTR* (International Bone Marrow Transplantation Registry); *MAGIC* (Mount Sinai Acute GvHD International Consortium)

a In the Minnesota criteria [19], overall grade III refers to liver = 2, 3 or 4; and/or GI = 2 or 3

b In the Minnesota criteria [19], overall grade IV refers to skin = 4; and/or GI = 4

**Table 3:** Comparison of the different guidelines available for chronic GvHD assessment: overall severity staging

	Original Seattle criteria [21]	Revised Seattle criteria [22]	NIH criteria (2005 [17] and 2014 [18])
Diagnosis			
	NA	NA	Based on either the presence of specific diagnostic signs or distinctive signs accompanied by additional confirmation (e.g. biopsy or other objective diagnostic test) in at least one target organ (skin & appendages, mouth, eyes, genitalia, esophagus, lungs and muscles & fascia)
Severity Scoring			
Limited	Limited skin AND/OR limited hepatic involvement	Limited skin AND/OR limited hepatic involvement OR single organ sicca syndrome (eyes, mouth, vagina)	Mild No more than two organs with a score^a^ of 1, except for lung
Extensive	Generalized skin involvement AND/OR major hepatic complications AND/OR an isolated sicca syndrome of the eyes, mouth AND/OR any other organ involvement	Generalized skin involvement AND/OR major hepatic complications AND/OR multiple organs involved (more than two, including “nails”), the presence of skin sclerosis/serositis or fasciitis, bronchiolitis obliterans, decreased performance status (<60% Karnofsky-Lansky index) or weight loss >15%	Moderate Any other severity scoring^a^ not included in the mild or severe categories
			Severe At least one organ with a score^a^ of 3 or a lung score^a^ of 2

*GvHD* graft versus host disease, *NA* not applicable, *NIH* National Institutes of Health

a Based on specific severity criteria described individually for the manifestations of chronic GvHD in eight target organs (skin & appendages, mouth, eyes, genitalia, GI tract, liver, lungs and muscles & fascia) and measured on a range of 0 (absent) to 3 (severe) for each organ [18]

**Table 4:** Suggested definitions for commonly used GvHD terminology

**Acute and chronic GvHD status**			
Clinical GvHD status	Acute or chronic GvHD inflammatory or worsening manifestations	GvHD sequelae^a^	Systemic immunosuppressive treatment
Active	Present	Irrelevant	Irrelevant
Controlled	Absent	Irrelevant	On immunosuppression or immunosuppression stopped for < 12^b^ to 24^c^ weeks
Inactive	Absent	Present	Off immunosuppression (immunosuppression stopped for > 12^b^ to 24^c^ weeks)
Resolved	Absent	Absent	Off immunosuppression (immunosuppression stopped for > 12^b^ to 24^c^ weeks)
**Acute and chronic GvHD onset**		
aGvHD onset		Timing post HCT or DLI
Classic	First episode of aGvHD^d^	≤Day 100
Late	First episode of aGvHD^d^	>Day 100
Recurrent	Recurrence of aGvHD^d^, after a period of aGvHD control, inactivity or resolution	>Day 100
Persistent	aGvHD^d^ signs persist beyond day 100 from a prior active classic aGvHD	>Day 100
cGvHD onset		Timing post HCT or DLI
De novo	First episode of cGvHD, without prior aGVHD	Irrelevant
Quiescent	Development of cGvHD, after a period of aGvHD control, inactivity or resolution	Irrelevant
Progressive	First episode of cGvHD, while aGvHD symptoms are still active	Irrelevant
	**Acute GvHD steroid response**	**Chronic GvHD steroid response**
Steroid refractoriness or resistance	Progression of aGvHD within 3–5 days of therapy onset with ≥2 mg/kg/day of prednisone **OR** failure to improve within 5–7 days of treatment initiation	cGvHD progression while on prednisone at ≥1 mg/kg/day for 1–2 weeks
	**OR** incomplete response after more than 28 days of immunosuppressive treatment including steroids	**OR** stable GvHD disease while on ≥0.5 mg/kg/day^e^ of prednisone for 1–2 months
Steroid dependence	Inability to taper prednisone below 2 mg/kg/day **OR** a recurrence of aGvHD activity during steroid taper	Inability to taper prednisone below 0.25 mg/kg/day^f^ in at least two unsuccessful attempts separated by at least 8 weeks
Steroid intolerance	Emergence of unacceptable toxicity due to the use of corticosteroids	

*DLI* donor lymphocyte infusion, *HCT* hematopoietic cell transplantation, *GvHD* graft versus host disease

a GvHD irreversible scars or fixed deficits

b For acute GvHD

c For chronic GvHD

d Presenting acute features only: maculopapular erythematous skin rash; and/or hyperbilirubinemia; and/or anorexia with weight loss, nausea, vomiting, diarrhea, severe abdominal pain, GI bleeding and/or ileus [16]

e Or 1 mg/kg every other day

f Or >0.5 mg/kg every other day
